# 

**Published:** 1853-04

**Authors:** 


### TO READERS AND CORRESPONDENTS.

Communications intended for publication, and all letters relating to the business of the Journal, or containing remittances in money, should be directed to Drs. Harris and Blandy, Baltimore, Md.

### *Medical and Dental Journals Received.*

1. *The Dental Register of the West.* Edited by James Taylor, M. D., D. D. S. Published by order of the Mississippi Valley Association of Dental Surgeons. January, 1853. *In Exchange.*
2. *The Dental News Letter.* January, 1853. *In Exchange.*
3. *Boston Medical and Surgical Journal.* Edited by J. V. C. Smith, M. D. January, February and March, 1853. *In Exchange.*
4. *The British and Foreign Medico-Chirurgical Review.* January, 1853. *In Exchange.*
5. *The American Journal of the Medical Sciences.* Edited by Isaac Hays, M. D., Surgeon to Wills Hospital, &c., January, 1853. *In Exchange.*
6. *The New York Dental Recorder.* Edited by C. C. Allen, M. D., Dentist, and A. Hill, D. D. S. February and March, 1853. *In Exchange.*
7. *The Ohio Medical and Surgical Journal.* Edited by Richard L. Howard, M. D. January, February and March, 1853. *In Exchange.*
8. *The Southern Medical and Surgical Journal.* Edited by J. P. Garvin, M. D. January, February and March, 1853. *In Exchange.*
9. *The New York Medical Gazette, and Journal of Health.* Edited by D. M. Reese, M. D., LLD. January, February and March, 1853. *In Exchange.*
10. *The New York Journal of Medicine and the Collateral Sciences.* Edited by S. S. Purple, M. D. January and February, 1853. *In Exchange.*
11. *The Medical Examiner.* Edited by F. G. Smith, M. D., and John Biddle, M. D. January, February and March, 1853. *In Exchange.*
12. *The Southern Journal of the Medical and Physical Sciences.* Edited by Drs. J. W. King, W. P. Jones, F. A. Ramsey, R. O. Currey and B. Wood. January and March, 1853. *In Exehange.*
13. *The New Jersey Medical Reporter, and Transactions of the New Jersey Medical Society.* Edited by Joseph Parish, M. D. January, February and March, 1853. *In Exchange.*
14. *The Western Lancet and Hospital Reporter.* Edited by L. M. Lawson, M. D., and George Mendenhall, M. D. Not received *In Exchange.*
15. *The North Western Medical and Surgical Journal.* Edited by J. Evans, M. D., and E. G. Meek, A. M., M. D. January, February and March, 1853. *In Exchange.*
16. *The Western Journal of Medicine and Surgery.* Edited by L. P. Yandell, M. D., and T. S. Bell, M. D. January, February and March, 1853. *In Exchange.*

17. *The New Hampshire Journal of Medicine.* Edited by EDWARD H. PARKER, A. M., M. D. January, February and March, 1853. *In Exchange.*

18. *The Charleston Medical Journal aud Review.* Edited by D. J. CAIN, M. D., and F. PEYREL PORCHER, M. D. October and November, 1852. *In Exchange.*

19. *The Buffalo Medical Journal.* Edited by AUSTIN FLINT, M. D. January, February and March, 1853. *In Exchange.*

20. *The Provincial Medical aud Surgical Journal.* Edited by G. W. H. RANKIN, M. D., and J. H. WALSH, Esq. January and February, 1853. *In Exchange.*

21. *The London Lancet,* Journal of British and Foreign, Surgical and Chemical Sciences, Chemistry, Literature and News. Editor THOMAS WAKELEY, M. D., for London. Sub-Editors, J. H. BENNET, M. D., and T. WAKELEY, M. R. C. S. E. December, 1852, January and March, 1853. *In Exchange.*

22. *The Northern Lancet and Gazette of Legal Medicine.* A monthly Journal of Medicine and General Science, Criticism, and Medical Jurisprudence. Edited by HORACE NELSON, M. D., and FRANCIS J. D. AVIGNON, M. D. January, February and March, 1853. *In Exchange.*

23. *The Dublin Medical Press.* January, February and March, 1853. *In Exchange.*

24. *Virginia Medical and Surgical Journal.* Edited by Drs. GEORGE A. OTIS and H. L. THOMAS. April, 1853.

---

*To Subscribers.*—We intend to publish on the cover of the next number of the Journal, a list of the names of those who have paid their subscriptions since the commencement of the present volume. We sent bills to most of our delinquents in January, and we are happy to say that very many promptly responded by enclosing to us the amount of their indebtedness; yet, very many are still in arrears. We trust, however, that ere our next issue they will all have remitted to us the amount of their indebtedness.

*Demonstration of Anatomy—being a Guide to the Knowledge of the Human Body by Dissection.* By George Viner Ellis, Professor of Anatomy in University College, London.

*A Manual of the Dissection of the Human Body.* By LUTHER HOLDEN, F. R. C. S., Demonstrator of Anatomy at St. Bartholomew's Hospital.

Both these works, London editions, can be obtained of Lindsay & Blakiston, book publishers, Philadelphia.

